# Professional Readiness of Senior Nursing Students: The Role of Professional Commitment and Values

**DOI:** 10.1111/nhs.70129

**Published:** 2025-08-03

**Authors:** Soner Berşe, Derya Tanriverdi, Ezgi Dirgar

**Affiliations:** ^1^ Department of Nursing, Faculty of Health Sciences Gaziantep University Gaziantep Turkey; ^2^ Department of Midwifery, Faculty of Health Sciences Gaziantep University Gaziantep Turkey

**Keywords:** nursing students, professional belonging, professional competence, professional readiness, professional values

## Abstract

Professional readiness is essential for newly graduated nurses to effectively transition into clinical practice and deliver quality patient care. This cross‐sectional study examined the factors influencing professional readiness among 206 senior nursing students at a public university in Southeastern Turkey. Data were collected using the Descriptive Information Form, Nursing Professional Commitment Scale (NPCS), Nursing Professional Values Scale (NPVS), and the Nursing Professional Readiness Perception Scale (NPRPS). Statistical analyses, including *t* tests, ANOVA, and path analysis, were conducted. Female students showed significantly higher levels of professional readiness and values compared to male students (*p* < 0.001). Income level and motivations for choosing nursing were also found to significantly impact readiness. On path analysis, professional commitment (*β* = 0.35, *p* < 0.05) and professional values (*β* = 0.349, *p* < 0.05) emerged as significant predictors of readiness, jointly explaining 34% (*R* ^2^ = 0.34) of its variance. These findings underscore the importance of fostering professional commitment and values in nursing education to support students' readiness for clinical roles and enhance professional development.

**Summary**

- Professional readiness among nursing students is significantly influenced by their commitment to the profession and internalization of professional values, highlighting the need to foster these attributes in nursing education.
- Female students demonstrated higher levels of professional readiness and values compared to their male counterparts, suggesting potential gender‐related differences in the development of professional identity.
- Sociodemographic and motivational factors, such as the voluntary choice of nursing as a career and satisfaction with nursing education, play a crucial role in shaping students' professional readiness and commitment.

## 1 Introduction

In health sciences and nursing disciplines, professional readiness is essential for ensuring safe, high‐quality, and patient‐centered care, ultimately contributing to better public health outcomes. While employers and academics may use varying terms and definitions to describe professional readiness, it is broadly recognized as an indicator of a student's potential for long‐term job performance and career development after graduation (Caballero and Walker 2010; Wang et al. 2024). It is crucial for graduating nursing students to have the necessary readiness to address the evolving healthcare needs as future providers. Mirza et al. (2019) argues that professional readiness plays a critical role in preparing newly graduated nurses for clinical practice and facilitating their integration into the healthcare system (Mirza et al. 2019). Furthermore, the professional readiness of newly graduated nurses contributes to the effectiveness of healthcare systems, especially in ensuring patient safety and improving clinical outcomes (Berşe et al. 2024).

*Nursing Professional Values* encompass the core ethical and moral principles that guide nurses' actions and decision‐making, including respect for human dignity, autonomy, accountability, and responsibility (Ayed et al. 2024). The *Perception of Professional Readiness* refers to nursing students' self‐assessment of their preparedness to fulfill clinical roles and apply theoretical knowledge in a real‐world healthcare environment (Tarhan and Yıldırım 2021). *Professional Commitment* reflects an individual's psychological attachment to the nursing profession, marked by belief in its goals and values, willingness to exert effort, and the desire to remain in the profession (Chen et al. 2025).

While the international literature includes numerous studies assessing nurses' and nursing students' perceptions of professional readiness or developing tools to measure professional readiness levels (Casey et al. 2011; Güner 2015; Jamieson et al. 2019; Leufer and Cleary‐Holdforth 2020; Woods et al. 2015), relatively few have explored the factors influencing this readiness. It is widely acknowledged that core factors such as clinical skills, theoretical knowledge, education, and academic achievement are the primary determinants of professional readiness. However, studies suggest that professional preferences, social intelligence, individual traits, organizational awareness, enjoyment of the profession, and the value placed on it also significantly affect professional readiness (Mirza et al. 2019; Suvedi et al. 2016; Walker et al. 2013). To address this, a methodological study in South Korea developed the Nursing Practice Readiness Scale (NPRS), a tool that assesses newly graduated nurses' readiness across several dimensions, including clinical judgment, professional attitudes, patient‐centered care, self‐regulation, and collaborative relationships. The validated scale offers a valuable framework for evaluating both the perceived competencies and the support needs of newly graduated nurses (Kim and Shin 2022).

Despite extensive research on nursing education and professional readiness, few studies have explored how professional values and sociodemographic variables shape students' perceptions of readiness. Addressing these gaps is crucial, as a deeper understanding of these dynamics could inform educational strategies aimed at fostering both professional commitment and values, ultimately improving nursing students' readiness for clinical practice. The present study offers important insights into these relatively underexplored relationships, particularly the interplay between professional identity and sociodemographic characteristics.

It is insufficient to evaluate a nurse's professional readiness solely in terms of technical knowledge and skills. A holistic approach that integrates both social and professional dimensions is needed. Accordingly, this study seeks to explore the factors affecting nursing students' readiness, particularly in relation to professional competence, sense of professional belonging, and professional values.

Professional commitment is a multidimensional concept that reflects an individual's physical, mental, and emotional connection to their profession. It includes belief in professional goals and values, a willingness to invest effort, a desire to remain in the profession, and an intrinsic appreciation for the work itself. This alignment between personal beliefs and professional determination can reduce turnover intentions, increase job satisfaction, and enhance the quality of patient care (Zhang et al. 2023; Zhao et al. 2022). Various factors, such as clinical learning environment, gender, and voluntary choice of profession, have been found to influence professional commitment (Wang et al. 2024; Ying et al. 2023; Zhang et al. 2023). Professional commitment plays a critical role in shaping nursing students' career decisions (Wang et al. 2024).

This study was designed to investigate the multifaceted factors that affect the professional readiness of nursing students, with a particular focus on professional competence, professional belonging, and professional values through an integrated and holistic perspective that includes both social and technical skill domains.

The following research hypotheses were tested in this study:

*Professional commitment is a significant positive predictor of nursing students' perception of professional readiness*.

*Professional commitment is a significant positive predictor of nursing students' professional nursing values*.

*Professional nursing values significantly and positively predict nursing students' perception of professional readiness*.

## 2 Methods

### 2.1 Study Design

This cross‐sectional study was conducted at the Faculty of Health Sciences of a public university located in Southeastern Turkey, between January and May 2023.

### 2.2 Sample

The study population comprised all final‐year students (*n* = 234) enrolled in the 8th semester of the Nursing Department at the Faculty of Health Sciences of a public university. The study aimed to include the entire student population; therefore, no sampling method was applied. A total of 206 students took part in the study, resulting in a participation rate of 88.03%. Although an a priori power analysis was not conducted during the initial stage of research planning, the high participation rate helped minimize sampling bias and enhanced internal validity.

All data collection procedures were carefully monitored to ensure the completeness of responses. Any questionnaires with missing or incomplete data were excluded from the analysis.

A post hoc power analysis conducted after data collection revealed an effect size of 0.380 and a statistical power of 88%, with a significance level (*α*) set at 0.05.

### 2.3 Data Collection

Data were collected in a classroom setting using Google Forms, a reliable online platform. The instruments used for data collection included the Descriptive Information Form, the Nursing Professional Commitment Scale, the Nursing Professional Values Scale, and the Perception of Professional Readiness Scale in Nursing.

#### 2.3.1 Descriptive Information Form

This form, developed by the researchers, consisted of 10 questions designed to gather sociodemographic information and insights related to the nursing profession and professional values (Caballero and Walker 2010; Ersoy and Ayaz‐Alkaya 2024; Gallen et al. 2019; Jamieson et al. 2019; Ningsih et al. 2024).

#### 2.3.2 Nursing Professional Commitment Scale (NPCS)

The NPCS, developed by Lu et al. (2000), assesses the professional commitment levels of nurses (Lu et al. 2000). The scale consists of 26 items distributed across three sub‐dimensions: willingness to make an effort, maintaining professional membership, and belief in goals and values. Nine items on the scale are reverse‐coded. The scale is a four‐point Likert‐type instrument, with total scores ranging from 26 to 104. The sub‐dimension scores range from 13 to 52 for willingness to make an effort, from 8 to 32 for maintaining professional membership, and from 5 to 20 for belief in goals and values. Higher scores indicate greater professional commitment (Lu et al. 2000). The original study reported an internal consistency of 0.94 (Lu et al. 2000). The validity and reliability of the Turkish version of the NPCS were demonstrated by Çetinkaya et al. (2015), who reported a Cronbach's *α* of 0.94 (Çetinkaya et al. 2015). In the current study, the Turkish version of the NPCS was used, which yielded a Cronbach's *α* of 0.88 and McDonald's *ω* coefficient of 0.88.

#### 2.3.3 Nursing Professional Values Scale (NPVS)

Developed by Darlene Weis and Mary Jane Schank, the NPVS is a five‐point Likert‐type scale (1 = *not important*; 5 = *extremely important*) comprising 44 items. The scale was designed to assess nurses' professional values in alignment with the American Nurses Association (ANA) code of ethics (Weis and Schank 2000). The total score is obtained by summing the responses, with scores ranging from 44 to 220. Higher scores indicate a greater emphasis on professional values and ethical considerations. The validity and reliability of the Turkish version of the NPVS were demonstrated by Orak and Alper who reported a Cronbach's *α* of 0.96 (Orak 2012). In the present study, the Turkish version of the NPVS was used, yielding a Cronbach's *α* of 0.96 and McDonald's *ω* coefficient of 0.96.

#### 2.3.4 Nursing Professional Readiness Perception Scale (NPRPS)

The NPRPS, developed by Tarhan and Yıldırım, consists of 15 items and three sub‐dimensions: professional adaptation (1, 2, 7, 8, and 9), communication and cooperation (3, 10, 11, 12, 13, 14, and 15), and professional competence (4, 5, and 6) (Tarhan and Yıldırım 2021). The scale uses a five‐point Likert‐type format and does not include negatively worded items. Scale and sub‐dimension scores are calculated by averaging the total score obtained from the entire scale or sub‐dimension. Scores range from 1 to 5 for both the whole scale and each sub‐dimension. A higher average score in a sub‐dimension indicates a higher perception of students' professional readiness in that domain. The original study reported a Cronbach's *α* of 0.90 (Tarhan 2020). In the current study, the scale demonstrated a Cronbach's *α* of 0.90 and McDonald's *ω* coefficient of 0.90.

### 2.4 Ethical Considerations

Approval for the study was obtained from the Clinical Research Ethics Committee of the affiliated university (Date: 2023, Decision No: 126), as well as the necessary permissions from the institution where the research was conducted. All participants were fully informed about the study's objectives and procedures, provided written consent, and were assured of their right to withdraw from the study at any time. Additionally, permission to use the scales was obtained from their respective owners via email. The study adhered to the ethical principles set forth in the Declaration of Helsinki throughout all stages of the research.

### 2.5 Data Analysis

The data were analyzed using SPSS version 22.0 and IBM AMOS version 24.0. Prior to the main analyses, the normality of data distribution was assessed using the Kolmogorov–Smirnov test. As the data met the assumptions of normality, appropriate statistical methods were employed, with the level of significance set at *p* < 0.05. Descriptive statistics, including counts, percentages, means, standard deviations, minimum, and maximum values, were computed and reported as mean ± SD, as well as frequency and percentage distributions.

To compare variables between two independent groups, the independent samples *t* test was applied. For comparisons involving more than two groups, one‐way analysis of variance (ANOVA) was used. Following significant ANOVA results, Tukey's Honestly Significant Difference (HSD) post hoc test was performed to determine specific group differences while controlling for Type I error. The assumption of homogeneity of variances was verified using Levene's test, which indicated that the variances were homogeneous across groups.

The adequacy of the sample size for the applied statistical techniques, including *t* tests, ANOVA, and path analysis, was confirmed. A post hoc power analysis yielded a statistical power of 88% (effect size = 0.38, *α* = 0.05), indicating that the sample was sufficiently powered to detect medium‐sized effects.

Path analysis was conducted to examine the hypothesized relationships among professional commitment, professional values, and perceived professional readiness. Parameter estimates for the path model were obtained using the maximum likelihood estimation method, which is appropriate for continuous data that are normally distributed.

The decision to employ path analysis was informed by the theoretical framework underpinning the study, which posits a sequential and mediated relationship among the core constructs. Specifically, it was hypothesized that professional commitment positively influences the development of professional values, which subsequently enhance students' perceptions of professional readiness. Path analysis facilitated the simultaneous evaluation of both direct and indirect pathways, offering a comprehensive understanding of the interconnected mechanisms through which these constructs contribute to the readiness of nursing students for professional practice.

## 3 Results

The mean age of the participants was 22.49 ± 1.16 years. The majority (84.95%) were female and had graduated from general high schools (94.20%). Regarding vocational preferences, 55.83% of the participants reported choosing nursing voluntarily, while 74.76% cited job security as their primary reason. Post‐graduation intentions varied, with 33.98% aiming to work in clinical settings and pursue postgraduate education. A large proportion (90.29%) considered nursing to be a professional career, and 63.11% had attended scientific meetings. The overall satisfaction rate among participants with their choice of profession was 73.79%. (Table 1).

**TABLE 1:** Sociodemographic characteristics of participants (*n* = 206).

Characteristic		*n*	%
Gender	Female	175	84.95
	Male	31	15.05
Type of high school Graduated	Health vocational high school	12	5.80
	General high school	194	94.20
Nationality	Turkish	197	95.63
	Other	9	4.37
Presence of healthcare professionals in your family or among your relatives	Yes	137	66.50
	No	69	33.50
Choosing the nursing profession voluntarily	Yes	115	55.83
	No	91	44.17
Your career plans after graduation	To become a clinical nurse	57	27.67
	Nursing without night shifts	33	16.02
	Becoming an academic	17	8.25
	Exploring a career in a field other than nursing	29	14.08
	Working in a clinic while pursuing postgraduate education	70	33.98
Reason for choosing the nursing profession	Academic career aspirations	20	9.71
	Job security	154	74.76
	Family influence	13	6.31
	Social status	19	9.22
Interest in membership in professional organizations	Yes	83	40.29
	No	123	59.71
Considering nursing as a professional career	Yes	186	90.29
	No	20	9.71
Participation in nursing‐related scientific meetings	Yes	130	63.11
	No	76	36.89
Satisfaction with being in the nursing department	Yes	152	73.79
	No	54	26.21

The senior nursing students' scores on the NPRPS, NPCS, and NPVS were examined. The total scores for the NPRPS ranged from 2.60 to 5.00, with a mean of 3.77 ± 0.50; the NPCS scores ranged from 51 to 103, with a mean of 73.47 ± 9.70; and for the NPVS, scores ranged from 83 to 155, with a mean of 112.44 ± 18.47 (Table 2).

**TABLE 2:** Scores of the students on the scales and subscales.

Scales and sub‐scales		Descriptive statistics		Cronbach's *α*
	*n*	$\bar{x}$	SD	
Nursing Professional Readiness Perception Scale (NPRPS)	**206**	**56.64**	**7.62**	**0.90**
Professional Adaptation	206	17.97	2.67	0.78
Communication and Cooperation	206	27.97	4.56	0.90
Professional Competence	206	10.71	1.78	0.69
Nursing Professional Commitment Scale (NPCS)	**206**	**73.47**	**9.70**	**0.88**
Willingness to make an effort	206	37.69	5.22	0.85
Maintaining professional membership	206	21.22	5.27	0.89
Belief in goals and values	206	14.56	2.12	0.68
Nursing Professional Values Scale (NPVS)	**206**	**3.62**	**0.59**	**0.96**
Human Dignity	206	3.65	0.67	0.92
Responsibility	206	3.54	0.63	0.87
Taking action	206	2.76	0.56	0.78
Security	206	3.75	0.67	0.82
Autonomy	206	3.67	0.59	0.85

*Note:* The bold values represent the total scores of the respective scales, distinguishing them from the subscale scores.

Gender differences were observed. Female students showed significantly higher professional readiness scores than male students (*t* = 3.244, *p* < 0.001). They also scored significantly higher on the NPCS (*t* = 1.978, *p* = 0.049). In terms of nationality, Turkish students had significantly higher NPVS scores compared to those of other nationalities (*t* = 2.197, *p* < 0.029).

Voluntary choice of the nursing profession was associated with significantly higher NPCS scores (*t* = 5.328, *p* = 0.001). Additionally, significant differences were observed in NPCS scores depending on the reasons for choosing nursing (*F* = 7.294, *p* < 0.05). Willingness to be a member of a professional nursing organization was significantly associated with both NPVS and NPCS scores (*t* = 1.909, *p* = 0.049 and *t* = 2.399, *p* = 0.017).

Participants who regarded nursing as a professional field reported significantly higher scores on both NPVS and NPCS (*t* = 3.379, *p* = 0.001 and *t* = 4.87, *p* = 0.001). Attendance at nursing‐related scientific meetings was also significantly associated with higher NPVS and NPCS scores (*t* = 3107, *p* = 0,002 and *t* = 4058, *p* = 0,001). Also, students who expressed satisfaction with their nursing education had significantly higher NPCS scores (*t* = 8.303, *p* = 0.001) (Table 3).

**TABLE 3:** Comparison of mean scale scores by selected student characteristics.

Characteristics		NPRPS	NPVS	NPCS
		$\bar{x}$ ± SD	$\bar{x}$ ± SD	$\bar{x}$ ± SD
Gender	Female	114.6 ± 18.71	57.08 ± 7.32	73.82 ± 9.88
	Male	102.75 ± 13.65	54.17 ± 8.91	71.55 ± 8.51
	*t*/*P*	**3.244/0.001** *	**1.978/0.049**	1.199/0.232
Type of high school graduated	Health vocational high school	117.42 ± 16.4	57.67 ± 5.15	66.59 ± 12.13
	Other	112.13 ± 18.59	56.58 ± 7.76	73.9 ± 9.41
	*t*/*p*	0.962/0.337	0.479/0.632	**−2.57/0.01** *
		b > a	b > a	
Nationality	Turkish	113.04 ± 18.67	57.16 ± 7.38	73.7 ± 9.84
	Other	99.34 ± 1.33	45.34 ± 2.7	68.56 ± 3.61
	*t*/*P*	**2.197/0.029** *	**4.787/0.001** *	1.561/0.12
Presence of healthcare professionals in your family or among your relatives	Yes	112.22 ± 18.64	58.76 ± 6.84	73.25 ± 10.01
	No	112.89 ± 18.26	52.44 ± 7.41	73.92 ± 9.11
	*t*/*P*	−0.246/0.806	**6.094/0.001** *	−0.464/0.643
Choosing the nursing profession voluntarily	Yes	112.46 ± 18.11	57.32 ± 7.18	76.48 ± 8.94
	No	112.42 ± 19.02	55.8 ± 8.12	69.68 ± 9.32
	*t*/*P*	0.013/0.989	1.426/0.155	**5328/0.001** *
Reason for choosing nursing	Academic career aspirations^(a)^	115.45 ± 9.33	56.25 ± 4.65	75.1 ± 7.52
	Job security^(b)^	111.9 ± 19.11	56.70 ± 7.93	73.23 ± 9.2
	Family influence^(c)^	117.85 ± 20.81	54.93 ± 8.48	64.54 ± 10.6
	Social status^(d)^	109.95 ± 19	57.79 ± 7.30	79.9 ± 10.65
	***F/P***	0.705/0.55	0.38/0.767	**7.294/0.001** * **a,d>c**
Interest in Membership in Professional Organizations	Yes	114.4 ± 19.67	57.87 ± 6.69	75.43 ± 10.59
	No	111.12 ± 17.58	55.82 ± 8.13	72.16 ± 8.85
	*t*/*p*	1.254/0.211	**1.909/0.049** *	**2.399/0.017** *
Considering nursing as a professional career	Yes	113.23 ± 18.53	57.22 ± 7.68	74.5 ± 9.34
	No	105.15 ± 16.63	51.3 ± 4.48	63.95 ± 7.78
	*t*/*p*	1.868/0.063	**3.379/0.001** *	**4.87/0.001** *
Participation in nursing‐related scientific meetings	Yes	113.9 ± 19.53	57.88 ± 7.66	75.5 ± 10.36
	No	109.95 ± 16.33	54.53 ± 7.15	70.02 ± 7.32
	*t*/*p*	1.484/0.139	**3.107/0.002** *	**4.058/0.001** *
Satisfaction with being in the nursing department	Yes	112.96 ± 18.32	57.11 ± 7.82	76.37 ± 8.48
	No	110.99 ± 18.98	55.34 ± 6.97	65.32 ± 8.2
	*t*/*p*	0.673/0.501	1.471/0.143	**8.303/0.001** *

*Note:* The bold values represent the total scores of the respective scales, distinguishing them from the subscale scores. Superscript letters (a, b, c, d) indicate significant differences between groups according to Tukey’s HSD post‐hoc test (*p* < 0.05). Groups that do not share the same letter differ significantly.

Abbreviations: *F* = one‐way ANOVA; *t* = ındependent samples *t* test.

* *p* < 0.05.

**FIGURE 1:**
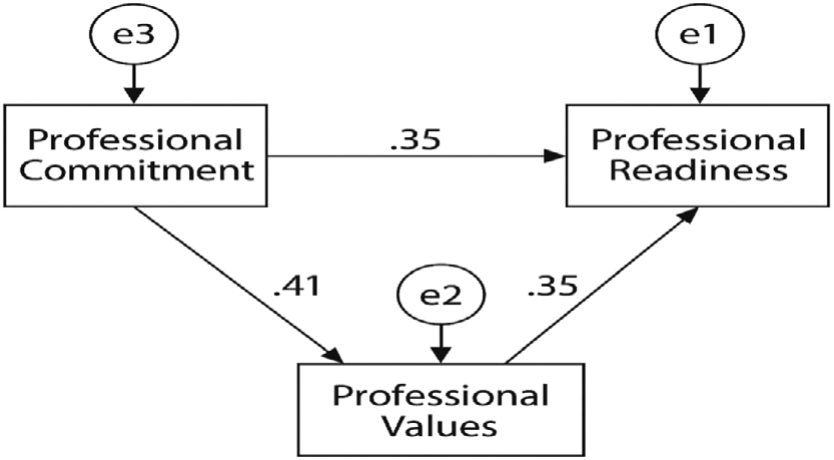
Path diagram of the proposed research model. This figure illustrates the hypothesized relationships among the three main constructs: Professional Commitment, Nursing Professional Values, and Perception of Professional Readiness. The model proposes both direct and indirect paths: Professional Commitment directly predicts both Professional Values (*β* = 0.41) and Perception of Readiness (*β* = 0.35); Professional Values also have a direct effect on Perception of Readiness (*β* = 0.35); The model explained 34% (*R* ^2^ = 0.34) of the variance in perception of readiness; Error terms (*e*_1_ and *e*_2_) represent the unexplained variance associated with dependent variables. All specified paths were statistically significant (*p* < 0.05), supporting the theoretical framework underlying the study.

The results of the path analysis revealed three statistically significant relationships within the proposed model (Table 4). Professional commitment demonstrated a significant moderate effect on professional values (*β* = 0.409, *p* < 0.05), as well as on perceived professional readiness (*β* = 0.35, *p* < 0.05). In turn, professional values showed a significant moderate effect on nurses' perception of professional readiness (*β* = 0.349, *p* < 0.05). Collectively, professional commitment and professional values explained 34% (*R* ^2^ = 0.34) of the variance in perceived professional readiness (Table 4). Figure 1 presents the structural path model that visually demonstrates these statistically significant relationships among professional commitment, professional values, and perceived professional readiness (Figure 1).

**TABLE 4:** Path analysis estimates for the proposed model.

Structural relationship			Standardized estimate (*β*)	Unstandardized estimate (*b*)	Standard error (SE)	Critical ratio (CR)	*p*
Professional Values	←	Professional Commitment	0.409	0.779	0.121	6.417	< 0.01
Perception of Professional Readiness	←	Professional Values	0.349	0.144	0.026	5.637	< 0.01
Perception of Professional Readiness	←	Professional Commitment	0.35	0.275	0.049	5.654	< 0.01

Model fit was assessed using the root mean square error of approximation (RMSEA), which was found to be 0.444. Although this value exceeds the conventional threshold (≤ 0.08), Doğan and Özdamar (2017) argue that reporting only RMSEA is sufficient for evaluating model fit, particularly in structural equation modeling applied to social and behavioral sciences. In line with this view, other fit indices such as CFI, GFI, and AGFI were not reported, as their simultaneous inclusion may lead to redundancy or inconsistent interpretations (Doğan and Özdamar 2017).

## 4 Discussion

This study investigated the interrelationships among senior nursing students' professional commitment, professional values, and perception of professional readiness, and examined how these constructs were influenced by various sociodemographic factors. The findings supported all three research hypotheses: professional commitment positively predicted both professional values and perceived professional readiness, and professional values also positively predicted perceived readiness. These results highlight the interconnected nature of these core professional constructs and emphasize their collective role in shaping nursing students' readiness for clinical practice.

Previous studies have explored professional commitment, professional values, and readiness across diverse nursing populations, including students (Arries 2020; Kara et al. 2022; Mukhtar et al. 2018) and clinical nurses (AlMekkawi and El Khalil 2020; Tehranineshat et al. 2020). In the present study, the total scores of the students on the NPRPS, HPVS, and NPCS were below the average, indicating a moderate level of professional readiness, values, and commitment.

One of the key findings of this study was the positive moderate effect of professional commitment on professional values. This suggests that nursing students who are more committed to their profession are more likely to adopt and internalize its core values. This is consistent with the findings of Peksoy et al. (2020), who reported that students with a strong sense of professional identity and internalization of professional values demonstrated greater professional commitment (Peksoy et al. 2020). Similarly, Özoğul and Eğe (2018) emphasized that individuals with higher professional commitment tend to identify more strongly with their profession and exhibit more positive attitudes towards it (Özoğul 2024). Professional commitment can be defined as an individual's intrinsic motivation towards fulfilling their professional role and responsibilities (Ayaz‐Alkaya et al. 2018). From this perspective, a high level of professional commitment may serve as a driving force behind the internalization of professional values. The current study also found that professional commitment had a direct positive and moderate effect on students' perceived professional readiness. This indicates that a stronger commitment to the profession enhances students' readiness for their future roles and responsibilities as professional nurses. In other words, as students' dedication to their chosen career path increases, their professional readiness to engage in professional practice also increases.

The present study found that the professional values of senior nursing students had a positive moderate effect on their perception of professional readiness. According to Bakır and Su (2022), professional values play a critical role in enhancing care outcomes, protecting professional identities, and shaping attitudes towards caregiving roles (Bakır and Su 2022). The path model explained 34% of the variance in professional readiness, which is considered acceptable for studies in social and educational sciences, where complex constructs are influenced by multiple variables (Hair et al. 2019). This result highlights the theoretical relevance of professional commitment and values, while also suggesting that other contextual or experiential factors—such as clinical learning, peer interaction, and educational satisfaction—may contribute to readiness. Nursing students who internalize these core values, such as ethics, responsibility, and respect for human dignity, are more likely to develop a stronger perception of professional readiness, thus enabling them to fulfill their professional roles more effectively and ethically. The combined effects of professional commitment and professional values explained 34% of the variance in professional readiness (*R* ^2^ = 0.34).

Several sociodemographic factors, including gender, school background, and profession‐related characteristics (e.g., reason for choosing nursing, membership in professional organizations, and future career intentions) were explored in this study to understand their influence on professional values, readiness, and commitment. In this study, female students showed higher levels of both professional readiness and professional values compared to their male counterparts. This aligns with findings from Karadağlı (2016), who reported higher scores on NPVS subdimensions of human dignity, responsibility, autonomy, and security among female nursing students (Karadağlı 2016). However, Ersoy and Ayaz‐Alkaya (2024) reported higher professional readiness among male students (Ersoy and Ayaz‐Alkaya 2024), suggesting that cultural factors and the professional identity of male nurses may influence their professional development. Male nurses have been part of the workforce in Turkey for approximately 15 years. The relatively recent integration of men into the nursing workforce, combined with prevailing societal perceptions of nursing as a female‐dominated profession, may contribute to lower professional identity awareness and perceived readiness among male nursing students. Although the gender difference in professional commitment was not significant in this study, female students exhibited higher mean scores than male students. This finding may reflect the stronger alignment of societal expectations and gender norms with the nursing profession for women, potentially leading to a deeper sense of identification with the profession and greater internalization of its values among female students.

In terms of nationality, domestic students demonstrated higher professional readiness and values compared to their foreign peers. This is not unexpected, considering that domestic students are typically embedded within the local culture and possess greater familiarity with the national healthcare system and practices. Foreign students, on the other hand, may face additional challenges that impact their professional readiness, as they are navigating their education in a different language, cultural context, and healthcare environment. Consistently, Ningsih et al. (2024) found no significant differences in professional readiness by ethnicity, but reported that domestic students showed stronger professional values compared to foreign students (Ningsih et al. 2024). Similarly, in a study involving Turkish, Spanish, and Tanzanian nursing students, Paşalak et al. (2021) reported that Turkish students scored higher on NPVS. However, the authors cautioned that cultural influences on professional values may vary not only across countries but also between different regions and educational institutions within the same country, limiting the generalizability of such findings (Paşalak et al. 2021).

The study also highlighted that the reasons for choosing the nursing profession influenced students' professional commitment. Students who chose nursing due to family pressure exhibited lower professional commitment, whereas those who selected the profession based on personal interest or academic and career‐oriented motivations reported higher commitment levels. Similarly, Zhang et al. (2023) found that Chinese nursing students who independently chose the profession demonstrated stronger professional commitment (Zhang et al. 2023). In contrast, those who were guided by family influence showed lower levels of commitment. Ekeng et al. (2022) also emphasized the substantial role parents play in shaping their children's career paths (Ekeng et al. 2022). Depending on parental values, education levels, and socioeconomic status, this influence may be either supportive or restrictive. Parents may unintentionally hinder their children's autonomy in career selection, potentially diminishing their long‐term satisfaction and commitment. Additionally, the current study revealed that having a family member in a healthcare profession positively influenced students' professional readiness. Having a family member with professional experience in healthcare may enhance students' awareness, interest, and understanding of the profession, serving as a role model and contributing to their readiness. To foster greater professional commitment, especially among students who choose nursing due to external pressures, targeted educational strategies and supportive interventions are essential.

Job dissatisfaction is one of the most common reasons for nurses to leave the profession (Cicolini et al. 2014). In this study, senior nursing students who reported satisfaction with their nursing education program demonstrated greater professional commitment. Similarly, it was reported that job satisfaction is positively correlated with professional commitment among both students and practicing nurses (Duran et al. 2021). These findings suggest that fostering satisfaction with the nursing education experience may play a crucial role in enhancing students' long‐term commitment to the profession. Therefore, promoting satisfaction during both education and clinical practice may serve as an effective strategy to strengthen professional commitment and, in turn, support organizational stability and retention.

### 4.1 Implications for Generalizability

While the results of this study offer valuable insights into the professional readiness of nursing students, it is essential to interpret these findings within the context in which they were obtained. As the study was conducted in a single public university in Southeastern Turkey, the extent to which the results can be generalized to other institutional, cultural, or educational contexts remains limited. Institutional environments, curricular structures, and cultural perceptions of the nursing profession may vary widely across regions and countries, potentially influencing professional commitment and readiness in different ways. Therefore, while the findings highlight critical associations, caution should be exercised when extrapolating them beyond the studied population. Future research should consider multi‐site or cross‐cultural studies to validate and expand upon these results in diverse educational and clinical settings.

### 4.2 Limitations

This study has several important limitations. While the study sample adequately represents the senior nursing students as the target population, caution should be exercised when attempting to generalize the findings to broader populations, as the research was conducted at a single institution in Southeastern Turkey. Furthermore, the model explained 34% of the variance in professional readiness (*R* ^2^ = 0.34), which reflects a moderate level of explanatory power in educational and behavioral research, but also suggests that other unexamined factors may contribute to professional readiness. It is also important to acknowledge that the path analysis conducted in this study estimates statistical associations between variables rather than causal relationships.

The cross‐sectional design of the study limits the ability to assess long‐term changes in students' professional readiness and professional values over time. As a result, the study cannot capture developmental trajectories throughout the course of nursing education.

Another limitation is the absence of an a priori power analysis. Although the study achieved a high participation rate and demonstrated sufficient post hoc power, future research would benefit from including power estimations during the planning stage to enhance methodological rigor. In addition, multicollinearity diagnostics such as variance inflation factor (VIF) and Tolerance values were not calculated, as the variables included in the path analysis model were theoretically distinct and exhibited only moderate correlations, suggesting a low risk of collinearity. Nevertheless, future studies employing regression‐based designs may consider including explicit multicollinearity testing.

## 5 Conclusion

This study contributes to the growing body of literature on nursing education by examining how professional commitment, professional values, and sociodemographic factors relate to perceptions of professional readiness among senior nursing students. While the path analysis confirmed the significant interrelationships among these core constructs, the findings also underscore the influence of gender, income level, and motivations for choosing the profession—factors often overlooked in existing research. By integrating these variables, the study highlights the multifaceted nature of professional readiness and the need to consider both personal and contextual elements in nursing education. The results may inform the development of more inclusive and responsive educational strategies that acknowledge student diversity and enhance readiness for clinical practice. Future research is encouraged to build on these findings by exploring additional factors and longitudinal outcomes that shape professional development in nursing students.

### 5.1 Relevance for Clinical Practice

This study highlights the critical role of professional commitment and values in shaping nursing students' readiness for clinical practice. By identifying these key factors, educators can implement targeted interventions that cultivate foster professional identity and better prepare students for the demands of the healthcare environment. Fostering professional values through structured educational programs can enhance ethical decision‐making, accountability, and respect for patient dignity, ultimately leading to improved patient care outcomes. Moreover, recognizing sociodemographic differences and motivations for pursuing the nursing profession can inform tailored strategies to support diverse student populations. Encouraging active participation in professional organizations and scientific activities can further strengthen students' sense of belonging and readiness, facilitating a smoother transition from education to clinical practice. These findings underscore the importance of adopting a holistic approach in nursing education to ensure that students are equipped not only with clinical skills but also with the professional values and commitment necessary to navigate the complexities of clinical settings effectively and ethically.

## Author Contributions

**Soner Berşe:** conceptualization, investigation, writing – original draft, methodology, writing – review and editing, data curation. **Derya Tanriverdi:** writing – review and editing, methodology, writing – original draft, supervision. **Ezgi Dirgar:** conceptualization, investigation, writing – original draft, validation, visualization, resources.

## Ethics Statement

The study was started after receiving the required permissions from the Non‐Interventional Research Ethics Committee (Date: 2023 Decision No: 126), Gaziantep University. We conducted according to the ethics guidelines set out in the Declaration of Helsinki. All the participant participating in the study were informed about the study, their written/verbal consents were taken, and they were also informed that they could leave the study at any time.

## Consent

Written informed consent was obtained from all participants, and all ethical procedures were followed in accordance with institutional guidelines and the Declaration of Helsinki.

## Conflicts of Interest

The authors declare no conflicts of interest.

## Data Availability

The data supporting the findings of this study are available from the corresponding author upon reasonable request.
